# The development of quality indicators for the prevention and management of postpartum haemorrhage in primary midwifery care in the Netherlands

**DOI:** 10.1186/1471-2393-13-194

**Published:** 2013-10-20

**Authors:** Marrit Smit, Susanne IC Sindram, Mallory Woiski, Johanna M Middeldorp, Jos van Roosmalen

**Affiliations:** 1 Department of Obstetrics, Leiden University Medical Centre, Albinusdreef 2, Leiden, 2300 RC, The Netherlands; 2 Department of Obstetrics, University Medical Centre St Radboud, Geert Grooteplein-Zuid 10, Nijmegen, 6500 HB, the Netherlands; 3 Department of Medical Humanities, EMGO Institute VU University Medical Center, van der Boechorststraat 7, Amsterdam, 1081 BT, The Netherlands

**Keywords:** Quality indicators, Postpartum haemorrhage, Midwifery, Delphi technique, Home birth, The Netherlands

## Abstract

**Background:**

At present, there are no guidelines on prevention and management of postpartum haemorrhage in primary midwifery care in the Netherlands. The first step towards implementing guidelines is the development of a set of quality indicators for prevention and management of postpartum haemorrhage for primary midwifery supervised (home) birth in the Netherlands.

**Methods:**

A RAND modified Delphi procedure was applied. This method consists of five steps: (1) composing an expert panel (2) literature research and collection of possible quality indicators, (3) digital questionnaire, (4) consensus meeting and (5) critical evaluation. A multidisciplinary expert panel consisting of five midwives, seven obstetricians and an ambulance paramedic was assembled after applying pre-specified criteria concerning expertise in various domains relating to primary midwifery care, secondary obstetric care, emergency transportation, maternal morbidity or mortality audit, quality indicator development or clinical guidelines development and representatives of professional organisations.

**Results:**

After literature review, 79 recommendations were selected for assessment by the expert panel. After a digital questionnaire to the expert panel seven indicators were added, resulting in 86 possible indicators. After excluding 41 indicators that panel members unanimously found invalid, 45 possible indicators were assessed at the consensus meeting. During critical evaluation 18 potential indicators were found to be overlapping and two were discarded due to lack of measurability.

**Conclusions:**

A set of 25 quality indicators was considered valid for testing in practice.

## Background

Postpartum haemorrhage (PPH), internationally defined as >500 mL of blood loss within 24 hours after childbirth, remains one of the leading causes of severe maternal morbidity and mortality worldwide, especially in low resource countries [1].

The definition of PPH, however, is not unified; in high-resource countries PPH is often defined as blood loss of at least 1000 mL, while a woman in good health can tolerate up to one litre of blood loss without showing early signs of shock [2-6]. Over the last 15 years, an increase in PPH has been observed in high-resource countries. The reasons for this remain unclear [7].

Almost one third of Dutch women (32.7%) give birth in ‘primary care’ which is low risk care supervised by a midwife (99% of births) or general practitioner (1% of births). Of all births in primary care, 64% occur at home [8].

In the Netherlands the overall prevalence of PPH (defined as >1000 mL blood loss), is 5.9%. Of all births in primary care, 3.4% is complicated with PPH [8]. When PPH occurs, women are referred to secondary care and treated by obstetricians. In a home birth setting, women are then transferred to hospital by ambulance. Audit of care provided in case of severe complications in pregnancy and childbirth has shown considerable room for improvement of PPH management [9,10].

The Dutch Society of Obstetrics and Gynaecology (NVOG) published guidelines concerning prevention and management of PPH for women giving birth in hospital supervised by an obstetrician [4]. At present, however, there are no guidelines on prevention and management of PPH in primary midwifery care in the Netherlands. Although published obstetrical guidelines can be and are used in primary midwifery care, the unique conditions in midwifery care (such as low-risk profile and birth at home) call for guidelines specifically designed for primary midwifery care.

The first step towards such guidelines is determining applicable items, preferably by using quality indicators. Quality indicators are derived from outcomes of studies, historical data and expert opinions and are defined as measurable elements of practice performance for which evidence or consensus exists. They can be used to assess and improve quality of care provided to the woman [11]. The aim of this study is to develop a set of quality indicators designed for the prevention and management of PPH in primary midwifery care.

## Methods

The RAND modified Delphi method was used to develop a set of quality indicators for prevention and management of PPH in primary midwifery care. This method has been proved valuable as a systematic method using current scientific evidence in conjunction with expert opinion [12-15]. For this study, ethical approval was not required.

### Indicator development procedure

**Figure 1:**
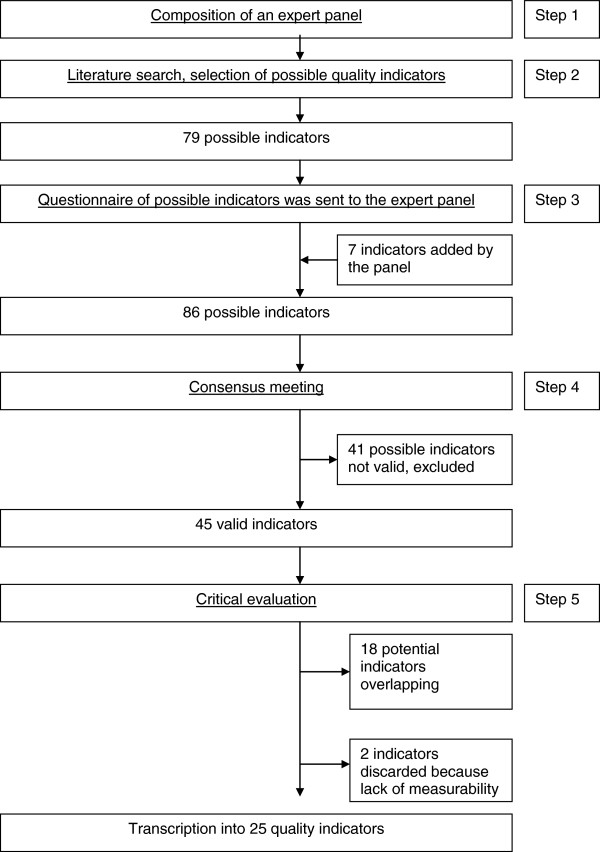
The process of quality indicator development according to the RAND-modified Delphi method for prevention and management of PPH in primary care in the Netherlands.

The procedure for quality indicator development consists of five steps: (1) composing an expert panel, (2) literature research and collection of recommendations, (3) questionnaire, (4) consensus meeting and (5), critical evaluation (Figure 1).

#### ***Step 1: composing an expert panel***

In order to capture all aspects of care concerning prevention and management of PPH, members were selected with expert knowledge in (at least) one of the following domains: primary midwifery care, secondary obstetric care, emergency transportation, maternal morbidity or mortality audit, quality indicator development or clinical guidelines development and representatives of professional organizations (Royal Dutch College of Midwives [KNOV] or NVOG).

#### ***Step 2: literature research and collection of possible indicators***

In order to identify possible indicators for PPH, first PubMed was searched using the following keywords: ‘postpartum haemorrhage’, ‘home birth’, ‘low-risk birth’, ‘prevention’ and ‘third stage of labour’ in combination with ‘guideline’ or ‘quality indicator’. The Internet was searched for reports and statements on PPH, especially in primary (midwifery) care. Following this, international guidelines, protocols and consensus statements were retrieved and collected. Indicators used in secondary obstetric care concerning prevention and management of PPH were included. Finally, in order to complete the preliminary set of possible indicators, manuals of obstetric emergency courses regarding prevention and management of PPH were studied. Due to the lack of a unified definition of PPH, PPH defined as 500 mL and 1000 mL were categorized separately. Some items, such as surgical procedures and embolisation, were clearly not applicable in primary care, and therefore deemed not relevant for this study.

Other items needed rephrasing for clarification of the possible indicator. The list of possible indicators was categorised into five domains: prevention, >500 mL blood loss <1000 mL, >1000 mL blood loss <2000 mL, >2000 mL blood loss and organization of care.

#### ***Step 3: questionnaire***

A questionnaire listing all possible indicators was sent to all panel members via e-mail. To facilitate decision-making, the source(s) and relevant literature citations for each potential indicator were provided.

Panel members were asked to score the possible indicators on a nine-point Likert scale, ranging from ‘one’, being a poor quality measure of care, to ‘nine’, being an excellent quality measure. In addition, panel members had the option of selecting ‘not assessable’. The respondents were asked to score each possible indicator with respect to their impact on both ‘health gain’ and ‘overall health efficacy’. Health gain was defined as: ‘An increase in the health of individuals or population’ and overall health efficacy was defined as: ‘prevention of unnecessary medical treatment and promotion of cost-effectiveness’ [14]. In addition, panel members were given the opportunity to provide comments or suggest additional indicators.

All data were collected and analysed using the Statistical Package for the Social Sciences (SPSS), version 17 (SPSS Inc., Chicago, IL, USA). The median panel rating and the amount of dispersion of ratings between panel members were calculated for each potential indicator. The comments and newly proposed quality indicators were collected. For an optimal assessment of all possible indicators offered to the panel, no indicators were discarded between this questionnaire round and the consensus meeting. All newly proposed possible indicators were added to the list. Overall agreement on each item was defined as 75% or more of ratings within a panel being in the lowest (1, 2, 3,) or the highest tertile (7, 8, and 9). The subsequent consensus meeting focused on indicators with low agreement.

#### ***Step 4: consensus meeting***

The expert panel was invited to a face-to-face consensus meeting. At the onset of the meeting, each panel member received the list of possible indicators, together with their own ratings from the questionnaire. The median rating and the frequency of responses for each possible indicator were also provided. Finally, panel members received the list of newly introduced potential indicators from step three. Individual ratings of the other panel members were kept confidential. Subsequently, the panel was divided into three groups of either four or five participants, every group consisting of at least one midwife and one obstetrician. Each group was assigned one or two domains (as described in step two), and were asked to evaluate the practical applicability of each possible indicator.

Each group (moderated by one of the authors) focused on indicators not unanimously agreed upon in the first questionnaire round. Indicators where the range of disagreement was widely spread were also discussed. The aim was to assess if there was genuine clinical disagreement about the validity of possible indicators or if there was a problem with phrasing. After the three groups assessed their assigned domains, the entire panel discussed potential indicators that were not agreed upon. After the panel meeting, the members were asked to rate all the indicators again. The final ratings were analysed in a similar manner as in step three. Analyses were performed based on the RAND/UCLA (University of California Los Angeles) appropriateness method [15]. An indicator was considered as ‘valid’ if there was an overall panel median score of eight or higher and if ‘agreement’ was reached between panel members.

#### ***Step 5: critical evaluation***

In adherence to the RAND method, the core panel critically evaluated the indicators with high agreement in step four. Emphasis was put on applicability, feasibility and measurability. Some indicators were modified or combined due to overlap between categories or pragmatic reasons concerning implementation, resulting in a final consensus-based set of indicators.

Each indicator was assessed and rephrased to define a numerator and denominator: the number of women in whom a specific test or intervention has been performed, divided by the number of women in whom this test or intervention should have been performed. By this last step use of the indicator can establish the percentage of adherence when evaluating quality of care.

## Results

The process of development of the indicators can be seen in Figure 1.

### Step 1

After selecting experts in one of the previously described domains, a panel of thirteen members was assembled consisting of five midwives (one of whom is first author), seven obstetricians (including three of the authors) and an ambulance paramedic. All midwives and obstetricians work in maternity care and are actively involved in at least one of the domains as described in the Methods section (Step 1).

### Step 2

A literature search resulted in a list of publications from which possible indicators could be extracted [2-4,9,10,16-30]. From these publications, all possible quality indicators for women at increased risk of PPH in secondary care were collected. More than half of the indicators were immediately discarded, as they are not applicable in primary midwifery care (e.g. surgical procedures and embolisation). Two studies on PPH and homebirth in an industrialised country were found. The authors made recommendations on referral in case of PPH and/or retained placenta after home birth [9,10]. These recommendations were incorporated in the list of possible indicators. This survey resulted in a list of 79 possible indicators, which were categorized into five domains as described in the ‘participants and methods’ section.

### Step 3

A questionnaire composed of the 79 possible indicators was sent to all panel members via email. The ambulance paramedic only rated possible indicators within his field of expertise and rated some indicators ‘not assessable’. The expert panel proposed seven additional possible indicators. Finally, a list of 86 possible indicators was prepared for assessment at the consensus meeting.

### Step 4

All panel members attended the meeting. After discussing and reassessing the 86 possible indicators, 45 recommendations were rated ‘valid’: four on prevention, nine on 500–1000 mL blood loss, 12 on >1000 mL blood loss, 14 on >2000 mL blood loss, and six on organization. The remaining 41 indicators were rated ‘not valid’ and subsequently excluded (Figure 1).

### Step 5

During critical evaluation by the core panel 18 potential quality indicators were found to be overlapping and two were discarded due to lack of measurability. Finally, a set of 25 potential indicators were transcribed into 25 quality indicators for prevention and management of PPH in primary midwifery care in the Netherlands (Table 1). The indicators each now contain a numerator and denominator, i.e. in case of PPH; the number of women with PPH who had an intravenous line is divided by the number of women with PPH.

**Table 1:** Final set of quality indicators for the measurement of PPH-care in primary care

		**Median *(n)***	**Agreement (% of panellists with score of 7, 8 or 9)**
	**For prevention of PPH, the midwife should;**		
1	Antenatally: identify elevated- or high risk of PPH and agree on preventive strategies*.^†^	8.5 *(12)*	100
2	At birth: identify elevated- or high risk of PPH and agree (or adjust) preventive strategies*.^†^	8 *(12)*	100
3	If high risk of PPH is assessed: have birth occur in hospital supervised by the obstetrician. ^†^	8.5 *(12)*	100
4	Routinely administer uterotonics (at least 5 IU oxytocin intramuscular). ^†^	9 *(12)*	83,3
	**In case of blood loss >500 mL, without signs of shock the midwife should;**		
5	Measuring blood loss by weighing. ^†^	9 *(12)*	91,6
6	Homebirth: in case of retained placenta; refer to secondary care after 30 minutes	9 *(13)*	92,3
7	Midwifery supervised hospital birth: in case of retained placenta; refer to secondary care after 30 minutes	9 *(13)*	75
8	Homebirth: if blood loss is not ceasing, refer to secondary care. ^†^	9 *(12)*	83,4
9	Midwifery supervised hospital birth: if blood loss is not ceasing, refer to secondary care. ^†^	9 *(12)*	83,3
10	Treat PPH as uterine atony (and apply bladder catheterization, uterine massage and oxytocin) until proven otherwise.	9 *(13)*	100
11	Post placental: if blood loss is not ceasing despite administration of uterotonics, examine for vaginal and perineal lesions. ^†^	7 *(12)*	75
	**In case of PPH of >1000 mL and/or signs of shock, the midwife should;**		
12	Inform the secondary caregiver (obstetrician).	9 *(13)*	100
13	Start an intravenous line and supply with fluids, using 0, 9% sodium chloride.	8 *(13)*	100
14	Monitor vital signs frequently (pulse, blood pressure, respiratory frequency).	8 *(13)*	92,4
15	Regardless of oxygen saturation, provide patient with 10–15 litre oxygen via non-rebreathing mask.	9 *(13)*	84,6
	**In case of PPH of > 1000 mL with signs of shock and/or >2000 mL blood loss the midwife should;**		
16	In case of persisting haemorrhage with signs of shock, perform uterine and/ or aortal compression. ^†^	8 *(12)*	83,3
17	Secure a second intravenous line (14 gauge).	9 *(13)*	79,9
18	If the patient has reduced consciousness due to hypovolemic shock, call for (paramedic) assistance in order to establish an open airway.	9 *(13)*	83,4
19	Immediately transfer patient to secondary care. ^†^	*(12)* Added in second round	100
	**Concerning cooperation and training;**		
20	Within every regional obstetric collaboration^£^ a regional PPH protocol should be present, based on national guidelines.	9 *(13)*	91,7
21	A regional PPH protocol should be the basis of regular audits.	9 *(13)*	83,3
22	The midwife is aware that ambulance transportation in case of PPH or retained placenta is always of the highest urgency category.	9 *(13)*	91,7
23	After each PPH with >2000 mL blood loss, the multidisciplinary team should debrief the situation.	8 *(13)*	83,4
24	Within the regional obstetric collaboration^£^ an annual training in obstetric emergencies should be provided.	9 *(13)*	100
25	In a homebirth situation, anticipation on possible ambulance transport is necessary; make sure the patient is at an accessible place for (all) caregivers in time.	9 *(13)*	100

* Preventative strategies imply consultation with an obstetrician to determine policy regarding PPH prevention e.g. birth supervised by obstetrician, or birth supervised by midwife, but in hospital with intravenous access prior to birth.

^£^ Regional obstetric collaboration; a quarterly meeting with obstetricians and midwifery practices within a region in the Netherlands where policy, collaboration and practical agreements are discussed.

^*†*^ The ambulance paramedic did not rate these items; it was not within his field of expertise and stated these as ‘not assessable’.

## Discussion

A RAND modified Delphi method approach was used to develop a set of 25 quality indicators. This is the first set of quality indicators concerning prevention and management of PPH in primary midwifery care in the Netherlands, to be used to assess care in case of PPH in primary care. This is an essential contribution to the development of guidelines of PPH in midwifery care.

The use of uterotonics, placing an intravenous line and quick referral in all cases of PPH were considered of great importance by the majority of the panel and thus incorporated in the final set. Possible indicators of the management in case of PPH > 2000 mL were either accepted or rejected with minimal dispersion. For some indicators however, assessment of validity was a source of discussion. For example, the routine use of oxytocin was hotly debated. As shown in a nationwide survey, most obstetricians consider this as part of standard care. In midwifery, though the use of uterotonics has increased over the last decade, this is no standard practice [30]. Currently, the Royal Dutch College of Midwives has not issued a guideline for women at low-risk of PPH or made any statement concerning management of third stage of labour. Also, in Dutch midwifery schools, no unambiguous policy is taught on the routine use of uterotonics. In the process of guideline development and implementation, routine use of uterotonics might be an item for further discussion, especially also because of the high prevalence of PPH in our country.

Although the effectiveness of comparable indicator development initiatives has been proven, there are limitations to this method [13,31,32]. Despite a thorough literature search, possible indicators may have been overlooked. However, the expert panel was given ample opportunity to propose additional items, both in the questionnaire round and during the consensus meeting. Of the seven additionally proposed indicators, three were incorporated in the final set [32]. It is well-documented that panel composition influences the outcome of the indicator-development process [33]. If more than one discipline of health care providers is included in an expert panel, lower agreement in rating between members are found, compared to when only expert in one discipline make up the panel. In this study, the panel consisted of a heterogenic group of professionals. Therefore, in case of high agreement, that indicator can be considered highly valid. Furthermore, it has been shown that the applied method (using a higher cut-off point for determining consensus with an overall median rate of 8 out of 9) enhances the reproducibility of ratings if a different set of panellists would rate the indicators [13].

In our literature search, many studies on PPH and homebirth originated in low-resource countries [19,24,34]. However, home birth in these countries is rarely a well-considered choice by women, and frequently being the result of poverty and lack of accessibility of health facilities. Therefore, it was often impossible to extrapolate recommendations into a western primary care setting. Only a few studies contain relevant information on home birth and referral in industrialised countries [9,35]. Thus, the scientific evidence base was limited in this area of primary care and necessitated the use of expert opinion in addition to available evidence. Due to this finding, we conclude that referral in case of PPH at home to hospital in industrialised countries is under-researched.

All quality indicators need to be validated, in order to ensure the clinical relevance [13,31]. Currently work is underway to validate this set by assessing collected cases of PPH in primary midwifery care in the Netherlands.

This set of indicators provides us with an instrument to assess the care commencing in a primary midwifery setting, before being transferred by ambulance to hospital.

## Conclusion

A set of 25 quality indicators for prevention and management of PPH in primary midwifery care in the Netherlands was developed. This is the first set of quality indicators which may serve as an assessment tool for prevention and management of PPH in primary care. This is of great interest, as the incidence of PPH is rising worldwide. Furthermore, existing guidelines for secondary care can be combined with these findings, so care throughout the care chain, including ambulance referral, can be thoroughly evaluated.

## Competing interests

The authors declare that they have no competing interests.

## Authors’ contributions

MS, SS, MW, AM and JvR planned the study. MS and SS prepared and carried out the Delphi procedure. MS, AM and JvR chaired the panel meeting. MS and SS conducted the data analyses and prepared the manuscript. JvR, MW and AM critical commented on the manuscript. All authors read and approved the final manuscript.

## Pre-publication history

The pre-publication history for this paper can be accessed here:

http://www.biomedcentral.com/1471-2393/13/194/prepub
